# 2′-5′-Oligoadenylate synthetase 1 polymorphisms are associated with tuberculosis: a case-control study

**DOI:** 10.1186/s12890-018-0746-x

**Published:** 2018-11-29

**Authors:** Shouquan Wu, Yu Wang, Guo Chen, Miaomiao Zhang, Minggui Wang, Jian-Qing He

**Affiliations:** 1 Department of Respiratory and Critical Care Medicine, West China Hospital, Sichuan University, No. 37, Guo Xue Alley, Chengdu, 610041 Sichuan People’s Republic of China; 2 Division of Geriatrics, Sichuan Provincial People’s Hospital, Chengdu, Sichuan China

**Keywords:** Alleles, Tuberculosis, 2′-5′-Oligoadenylate synthetase 1, Polymorphism, Association study

## Abstract

**Background:**

2′-5′-Oligoadenylate synthetase 1 (OAS1) plays an important role in inflammatory immune reactions. *OAS1* polymorphisms have been associated with increased susceptibility to various diseases. We investigated the association of polymorphisms in *OAS1* with tuberculosis (TB).

**Methods:**

A total of 1215 TB cases and 1114 healthy controls were enrolled from two independent studies. Genotyping was conducted using the improved multiplex ligase detection reaction (iMLDR) method. Associations between *OAS1* polymorphisms (rs2240190, rs1131454, 10,774,671 and 11,066,453) and TB risk were established based on distributions of allelic frequencies using different genetic models.

**Results:**

Significant association was observed between rs10774671, rs1131454 and TB. In the initial study, the G allele of rs10774671 was a significantly protective factor against TB (*P* = 0.006) and the genotype of GG differed significantly between TB patients and controls under the codominant model (*P* = 0.008) after Bonferroni correction. In the validation study, we also observed that the rs10774671 G allele (*P* = 0.001) and GG genotype (*P* = 0.001) were associated with TB. In addition, we found that the rs1131454 G allele (*P* = 0.004) and GG genotype (*P* = 0.001) were protective against TB in the Chinese Han population.

**Conclusions:**

We report novel associations of polymorphisms in *OAS1* with TB in the Chinese Tibetan and Han populations. Similar studies in different populations and functional studies are warranted to confirm our results.

## Introduction

Tuberculosis (TB) is an infectious disease that constitutes a major global health problem. It is a major cause of morbidity and mortality globally, particularly in Asia and Africa, and ranks alongside human immunodeficiency virus (HIV) as a leading cause of death worldwide [1]. In 2017, an estimated 1.7 billion individuals were newly infected with the causative agent of TB [1]. However, only 5–15% of them will develop TB during their lifetime [2]. The outcome of TB infection is affected by many factors, such as malnutrition, co-infection with other pathogens, exposure to microbes and previous vaccination [3]. It was reported that host genetic factors play a crucial role in determining an individual’s susceptibility to TB [4]. Recently, a number of genes potentially associated with TB susceptibility have been analysed in case-control studies.

Studies performed in mouse models and in patients indicate an important role of interferon (IFN) during TB infection [5, 6]. IFN-α, IFN-β and IFN-γ play significant immunomodulatory roles in the protection of the host against infections. As the main mediator of the type II immune response, IFN-γ is critical for controlling infections by intracellular pathogens, such as Mycobacteria [7]. Specifically, IFN-γ plays a vital role in resistance to *Mycobacterium tuberculosis* (*MTB*) by activating macrophages, monocytes and Th1 cells [8]. 2′-5′-oligoadenylate synthetases (OASs) are IFN-inducible enzymes that play vital roles in the innate immune response against viruses. The OAS family includes OAS1, OAS2, OAS3, and OAS-like (OASL) protein. The *OAS1* gene encodes OAS1, which is an extensively characterized enzyme induced by IFNs [9]. Binding of IFNs to their specific receptors results in induction of *OAS1* gene expression [10]. OAS1 is activated by the presence of double-stranded RNA and stimulates the oligomerisation of ATP into 2′,5′-linked oligoadenylates (2-5A) [11]. 2-5A can bind to latent ribonuclease L (RNase L), which then dimerizes into the active form. This active form facilitates apoptosis [12], attenuates proliferation [13] and inhibits protein synthesis [13]. It is possible that polymorphisms in *OAS1* influence the expression of IFN-γ, the elimination of *MTB*, and ultimately affect the development of TB [14, 15].

Other OAS family members including OAS2, OAS3, and OASL also have been the focus of much research. OAS2 encodes the p69 and p71 IFN-induced isoforms [16]. OAS2 play an important role in the control of bacterial infection through direct interaction with pattern-recognition receptors. It also has immunomodulatory function through immune cell receptor interaction [16]. In terms of the ability to synthesize 2-5A, OAS3 shows higher activity than OAS1 [17]. OAS3 is specialized for binding long dsRNA. The major function of OAS3 during infection is to produce 2-5A activators of RNaseL [18]. In contrast to other OAS family members, OASL lacks any synthetic activity. OASL has the ability to regulate type I IFN responses when infected with pathogens. It has been suggested that OASL could act as a regulator in the control of antiviral innate immunity through IFN signalling [19].

Single-nucleotide polymorphisms (SNPs) can regulate *OAS1* function at multiple levels, including expression, alternative splicing and enzyme activity. So far, among the OAS gene cluster, *OAS1* polymorphisms have been the most studied and were reported to influence susceptibility to various diseases. For example, SNPs in *OAS1* have been identified as candidates for susceptibility to viral infections, such as West Nile virus [20], hepatitis C [21], Chikungunya [22], dengue [23] and measles [24]. Previous data also indicated that *OAS1* polymorphisms and haplotypes were potential risk factors for autoimmune conditions, including type 1 diabetes [25] and multiple sclerosis [26]. In addition, *OAS1* polymorphisms were reported to be associated with respiratory infection [27]. However, no previous studies have examined the association between *OAS1* and TB.

Collectively, these observations prompted us to propose the hypothesis that *OAS1* polymorphisms confer susceptibility to TB. The purpose of the study was to evaluate the prevalence of polymorphisms within the *OAS1* gene in TB cases and healthy controls from the Chinese Tibetan and Han populations.

## Methods

### Population study

The initial study comprised 613 patients with TB and 602 healthy controls recruited from the People’s Hospital of the Aba Tibetan Autonomous Prefecture. In the independent validation study, we selected 571 TB cases and 543 healthy controls based on the same inclusion and exclusion criteria from the Chinese Han population. All subjects were unrelated ethnic Chinese.

The patients’ selection criteria were: 1) written consent and agreement to participate the study; 2) having a diagnosis of TB; 3) age ≥ 18 years. The diagnosis of TB was based on sputum smear tests, sputum culture, clinical symptoms and radiological and histological pathologic examinations. Patients with HIV, hepatitis B or C, diabetes, immune-mediated disorders or other lung diseases were excluded from our study. Controls were individuals without history of TB and active TB infection that had undergone routine medical examination.

Informed consents were obtained from all individual participants included in the study. The study was approved by the ethical committee of the West China Hospital of Sichuan University.

### SNP selection and genotyping

Tag SNPs in the study were downloaded from the Chinese Han in Beijing database of HapMap (http://hapmap.ncbi.nlm.nih.gov/index.html.en, HapMap Data Rel 27 Phase II + III, on NCBI B36 assembly, dbSNP b126) in a region stretching from 1000 base pairs upstream and 1000 base pairs downstream of *OAS1*; other SNPs of *OAS1* were also selected from previous studies [27, 28]. Tag SNPs were filtered according to the following criteria: (i) Hardy-Weinberg equilibrium (HWE) test *P* ≥ 0.05; (ii) r^2^ of pairwise linkage disequilibrium (LD) ≤ 0.8 and (iii) minor allele frequency (MAF) ≥ 0.05.

A sample of 5 ml of peripheral blood was obtained from each participant and placed in a test tube containing EDTA. We then isolated genomic DNA from the collected whole blood samples using a genomic DNA purification kit, in accordance with the manufacturer’s instructions (Axygen Scientific Inc., Union City, CA, USA). Genotyping was conducted using PCR and the improved multiple ligase detection reaction (iMLDR). Five percent of samples were re-genotyped to verify the initial results.

### Statistical analysis

HWE and the differences in sex between the case and control were evaluated by the Chi-squared test. Unpaired *t*-tests were used to evaluate the significance of differences in age between the case and control groups. Genotype and allele frequencies of SNPs in the TB group vs. those in the control group were calculated by logistic regression. As the genetic determinants of TB were age- and gender-dependent [29, 30], logistic regression was used to adjust for gender and age. Odds ratio (OR) and 95% confidence interval (95% CI) were examined to estimate the magnitude of the risk. LD and haplotype analyses were conducted using the SHEsis online software platform (http://analysis.bio-x.cn). A 2-sided *P* value of less than 0.05 was considered statistically significant and *P* values were conservatively Bonferroni-corrected for multiple comparisons. Power calculation was performed (*α =* 0.05) by Quanto software (version 1.2.4, May 2009). The power of the two cohorts to detect relative risks (RR) of 2.0, 3.0 and 4.0 using the four SNPs studied under an allele genetic model were estimated by the Quanto software and are listed in Table S1. The two cohorts have reasonable power (> 80%) to detect genetic factors with RR 2.0 and above under the allele model in all four SNPs (Table S1). Gene-gene interaction was detected using the multifactor dimensionality reduction (MDR) software and MDR permutation testing module (http://www.epistasis.org and http://sourceforge.net/projects/mdr/files/mdrpt). We performed an eQTL analysis by using the GTEx Portal database (https://gtexportal.org/home/). All analyses were conducted using the Statistical Package for the Social Sciences (SPSS; SPSS Inc., Chicago, IL, USA), version 19.0.

Genotype data of rs10774671 in the Han Chinese in Beijing (CHB) were obtained from the HapMap project and whole-genome Expression data were obtained from the Gene Expression Omnibus of PubMed. rs10774671 genotype data were downloaded from the 1000 Genomes Browser (https://www.ncbi.nlm.nih.gov/variation/tools/1000genomes/). DNA samples were collected from participants living in the residential community at Beijing Normal University. As a result, a total of 40 individuals provided genotypic data of rs10774671. We then used the mRNA data obtained from lymphoblastoid cell lines (extracted from Gene Expression Omnibus of Pubmed, accession number GSE6536) to analyze the associations between rs10774671 and *OAS1* mRNA expression levels [31].

## Results

### Demographic characteristics of the study groups

In the initial study, the number of cases was 613 (male = 392; female = 221) and the controls was 602 (male = 392; female = 210). The validation cohort comprised 584 cases (male = 299; female = 285) and 543 controls (male = 266; female = 277). The mean age was 34.53 ± 14.54 years for the case group and 34.63 ± 13.85 years for the control group in the initial study. There were no significant differences between the case and control groups regarding age and sex (*P* > 0.05) in this cohort. However, the mean age was significantly different between cases (mean age = 36.50 ± 15.49) and controls (mean age = 39.35 ± 15.13) in the validation cohort (*P* = 0.002).

### Analysis of HWE and association of *OAS1* SNPs with TB

A total of four SNPs (rs2240190, rs1131454, 10,774,671 and 11,066,453) were selected for analysis. Two of these four were tag SNPs (rs1131454 and rs10774671). All four SNPs did not deviate from HWE. The MAFs of SNPs were more than 0.05. Table 1 provides basic information on the four SNPs in our study.

**Table 1:** Characteristics of the 4 SNPs in *OAS1* in the present study

SNP	Position	Region	Tibetan population		Han population	
			MAF	HWE	MAF	HWE
rs2240190 (C > A)	113,346,127	Intron 1	0.190	0.975	0.190	0.783
rs1131454 (A > G)	113,348,870	Exon 3	0.406	0.973	0.379	0.924
rs10774671 (A > G)	113,357,193	Exon 7 splice acceptor site	0.237	0.294	0.210	0.819
rs11066453 (A > G)	113,365,621	Intron 7	0.136	0.807	0.132	0.848

*Abbreviation*: *SNP* single nucleotide polymorphism, *MAF* minor allele frequency, *HWE* Hardy Weinberg equilibrium

The allele frequencies in the two groups are shown in Table 2. The genotype frequencies of the *OAS1* polymorphisms under different genetic models are summarized in Table 3. In the initial cohort, a significant association was only detected in the allele frequency of rs10774671 and the G allele acted as a protective factor against TB (*P* = 0.006, OR = 0.74, 95% CI: 0.59–0.92). Compared with the AA genotype, the frequency of the GG genotype of rs10774671 polymorphism in the case group significantly differed from the controls under the codominant model (GG vs. AA: *P* = 0.008, OR = 0.41, 95% CI: 0.21–0.80), suggesting that rs10774671 decreased the risk of TB.

**Table 2:** Allele distribution of *OAS1* polymorphisms in the two populations

SNP	Tibetan population				Han population			
	Cases, n (%)	Controls, n (%)	*P* ^#^	OR^#^(95%CI)	Cases, n (%)	Controls, n (%)	*P* ^#^	OR^#^(95%CI)
rs2240190(C > A)								
C	941(76.8)	954(79.2)			918(81.0)	864(80.0)		
A	285(23.2)	250(20.8)	0.128	1.16(0.96–1.41)	216(19.0)	216(20.0)	0.556	0.94(0.76–1.16)
rs1131454(A > G)								
A	778(63.5)	726(60.3)			704(62.1)	610(56.5)		
G	448(36.5)	478(39.7)	0.110	0.88(0.74–1.03)	430(37.9)	470(43.5)	0.004*	0.78(0.66-0.93)
rs10774671(A > G)								
A	1051(85.7)	984(81.6)			896(79.0)	793(73.4)		
G	175(14.3)	222(18.4)	0.006*	0.74(0.59-0.92)	238(21.0)	287(26.6)	0.001*	0.72(0.59-0.87)
rs11066453 (A > G)								
A	1171(95.5)	1124(93.4)			984(86.8)	937(86.8)		
G	55(4.5)	80(6.6)	0.022	0.66(0.47–0.94)	150(13.2)	143(13.2)	0.807	1.03(0.81–1.32)

*SNP* single nucleotide polymorphism, *CI* confidence interval, *OR* odds ratio

^#^adjusted by sex and age

*Bonferroni correction was performed with *p* ≤ 0.0125 (0.05/4) considered significant

**Table 3:** Association between genotype of *OAS1* and TB in the two populations

SNP	Genetic model	genotype	Tibetan population				Han population			
			Case, n (%)	Controls, n (%)	*P* ^*#*^	OR^#^(95%CI)	Case, n (%)	Controls, n (%)	*P* ^*#*^	OR^#^(95%CI)
rs2240190 (C > A)	Codominant	CC	361(58.9)	383(63.6)	–	1.00	368(64.9)	343(63.5)		
		CA	219(35.7)	188(31.2)	0.084	1.24(0.97–1.58)	182(32.1)	178(33.0)	0.659	0.94(0.73–1.22)
		AA	33(5.4)	31(5.1)	0.567	1.16(0.70–1.94)	17(3)	19(3.5)	0.637	0.87(0.44–1.69)
	Dominant	AA+CA	252(41.1)	219(36.3)	0.084	1.23(0.97–1.55)	199(35.1)	197(36.5)	0.595	0.94(0.73–1.20)
		CC	361(58.9)	383(63.6)	–	1.00	368(64.9)	343(63.5)		
	Recessive	AA	33(5.4)	31(5.1)	0.819	1.06(0.64–1.76)	17(3)	197(36.5)	0.673	0.87(0.44–1.69)
		CC + CA	580(94.6)	571(94.9)	–	1.00	550(73.4)	521(96.5)		
rs1131454 (A > G)	Codominant	AA	246(40.1)	230(38.2)	–	1.00	205(36.2)	170(31.5)		
		GA	286(46.7)	266(44.2)	0.971	1.01(0.79–1.28)	294(51.9)	270(50.0)	0.353	0.88(0.68–1.15)
		GG	81(13.2)	106(17.6)	0.054	0.71(0.51–1.01)	68(12.0)	100(18.5)	0.001*	0.52(0.36-0.76)
	Dominant	GG + GA	367(59.9)	372(61.8)	0.484	0.92(0.73–1.16)	362(42.0)	370(68.5)	0.067	0.79(0.61–0.02)
		AA	246(40.1)	230(38.2)	–	1.00	205(36.2)	170(31.5)		
	Recessive	GG	81(13.2)	106(17.6)	0.037	0.72(0.52–0.98)	68(12.0)	100(18.5)	0.002*	0.59(0.42-0.82)
		AA+GA	522(86.8)	496(82.4)	–	1.00	499(88.0)	440(81.5)		
rs10774671 (A > G)	Codominant	AA	451(73.6)	409(68.0)	–	1.00	351(61.9)	294(92.4)		
		GA	149(24.3)	164(27.2)	0.148	0.83(0.64–1.07)	194(34.2)	205(38.0)	0.053	0.78(0.61–1.00)
		GG	13(2.1)	29(4.8)	0.008*	0.41(0.21-0.80)	22(3.9)	41(7.6)	0.001*	0.40(0.23-0.69)
	Dominant	GG + GA	162(26.4)	193(32.0)	0.032	0.76(0.59–0.98)	216(28.4)	246(45.6)	0.007	0.72(0.56–0.91)
		AA	451(73.6)	409(68.0)	–	1.00	351(61.9)	294(92.4)		
	Recessive	GG	13(2.1)	29(4.8)	0.013	0.43(0.22–0.83)	22(3.9)	41(7.6)	0.005*	0.46(0.27-0.79)
		AA+GA	600(97.9)	573(95.2)	–	1.00	545(96.1)	499(92.4)		
rs11066453 (A > G)	Codominant	AA	558(91.0)	525(87.2)	–	1.00	426(75.1)	408(75.6)		
		GA	55(9.0)	74(12.3)	0.060	0.70(0.49–1.02)	132(23.3)	121(22.4)	0.550	1.09(0.82–1.45)
		GG	0(0)	3(0.5)	–	–	9(1.6)	11(2.0)	0.592	0.78(0.32–1.92)
	Dominant	GG + GA	55(9.0)	77(12.8)	0.035	0.68(0.47–0.97)	141(20.2)	132(24.4)	0.649	1.07(0.81–1.41)
		AA	558(91.0)	525(87.2)	–	1.00	426(75.1)	408(75.6)		
	Recessive	GG	0(0)	3(0.5)	–	–	9(1.6)	132(24.4)	0.558	0.77(0.31–1.87)
		AA+GA	613(100)	599(99.5)	–	–	558(98.4)	529(98.0)		

*SNP* single nucleotide polymorphism, *CI* confidence interval, *OR* odds ratio

^#^adjusted by sex and age

*Bonferroni correction was performed with p ≤ 0.0125 (0.05/4) considered significant

To verify the results of the Tibetan population, we analyzed an independent population of Chinese Han individuals. We found that rs10774671 was also significantly associated with TB in the Chinese Han population. Compared with allele A, the G allele was more common in the control group (*P* = 0.001, OR = 0.72, 95% CI: 0.59–0.87). We also found that the GG genotype was a protective factor against TB under codominant (GG VS. AA: *P* = 0.001, OR = 0.40, 95% CI: 0.23–0.69) and recessive (GG VS. AA+GA: *P* = 0.005, OR = 0.46, 95% CI: 0.27–0.79) models (Table 4). In addition, rs1131454 was associated with TB susceptibility in the validation study. Compared with the control group, the TB group had lower proportions of the G allele (*P* = 0.004, OR = 0.78, 95% CI: 0.66–0.93) and GG genotype (codominant model: GG vs. AA, *P* = 0.001, OR = 0.52, 95% CI: 0.36–0.76; recessive model: GG vs. AA+GA, *P* = 0.002, OR = 0.59, 95% CI: 0.42–0.82).

**Table 4:** Haplotype analyses in the Tibetan population

	Tibetan population				Han population			
Haplotype	Cases, n (%)	Controls, n (%)	P	OR	Cases, n (%)	Controls, n (%)	P	OR
AGAA	198 (16.1)	162 (13.5)	0.038	1.27(1.01–1.59)	132(11.4)	113(10.4)	0.454	1.11 (0.85–1.45)
AGGA	53 (4.3)	80 (6.6)	0.018	0.65(0.46–0.93)	74(6.4)	94(8.7)	0.043	0.72(0.53–0.99)
CAAA	690 (56.3)	640 (53.2)	0.035	1.19(1.01–1.40)	538(46.4)	459(42.5)	0.052	1.18(1.00–1.40)
CAAG	54 (4.4)	78 (6.5)	0.034	0.68(0.48–0.97)	150(12.9)	142(13.1)	0.926	0.99(0.77–1.27)
CGAA	75 (6.1)	94 (7.8)	0.153	0.80(0.58–1.09)	60(5.2)	69(6.4)	0.227	0.80(0.56–1.15)
CGGA	121 (9.9)	141 (11.7)	0.207	0.85(0.66–1.10)	190(16.4)	193(17.9)	0.357	0.90(0.72–1.12)
Other^a^	35 (2.9)	9 (0.7)	–		15(1.4)	11(0.9)		

*CI* confidence interval, *OR* odds ratio

^a^Those lowest frequency threshold (LFT) < 0.03 were pooled in this part

Bonferroni correction was performed with p ≤ 0.008 (0.05/6) considered significant

For each haplotype, alleles are arranged in order of rs2240190, rs1131454, 10,774,671 and 11,066,453

### Linkage disequilibrium and haplotype analyses

**Fig. 1:**
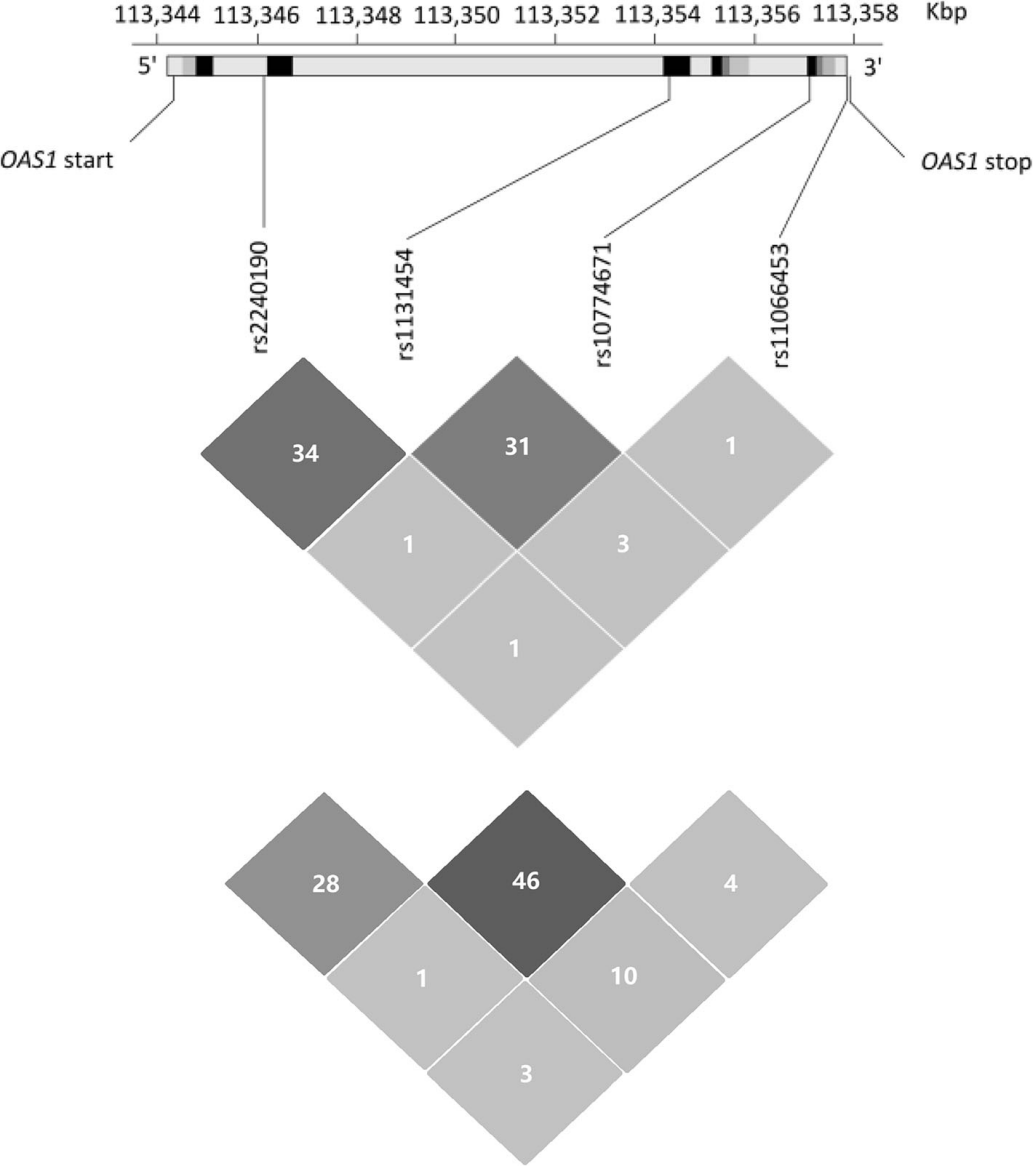
Genomic structure, localization and LD of the genotyped polymorphisms within *OAS1* in both Tibetan (above) and Han (below) populations. The figure shows the genomic structure and localization of the genotyped polymorphisms in *OAS1* retrieved from the HapMap database. In the upper panel, the black and grey boxes represent exons, and light grey ones represent introns. Locations of the four genotyped SNPs are indicated by the connecting line. In the lower panel LD (r^2^ value) estimates corresponding to all pairs of SNPs are shown.

Information on LD for these four SNPs in the Chinese Tibetan population is shown in Fig. 1. LD among these four SNPs in the *OAS1* gene was evaluated by the LD test using r^2^, which confirmed that these SNPs were not in LD (r^2^ < 0.5). No haplotypes were associated with TB in the two studies (Table 4).

### Gene–gene interactions

rs2240190, rs1131454 and rs10774671 formed the best gene-gene interaction model with a testing balanced accuracy of 57.61% and cross-validation consistency of 10/10. It was confirmed statistically significant using 1000-fold permutation testing (*P* = 0.001).

### eQTL and gene expression analysis for rs10774671

**Fig. 2:**
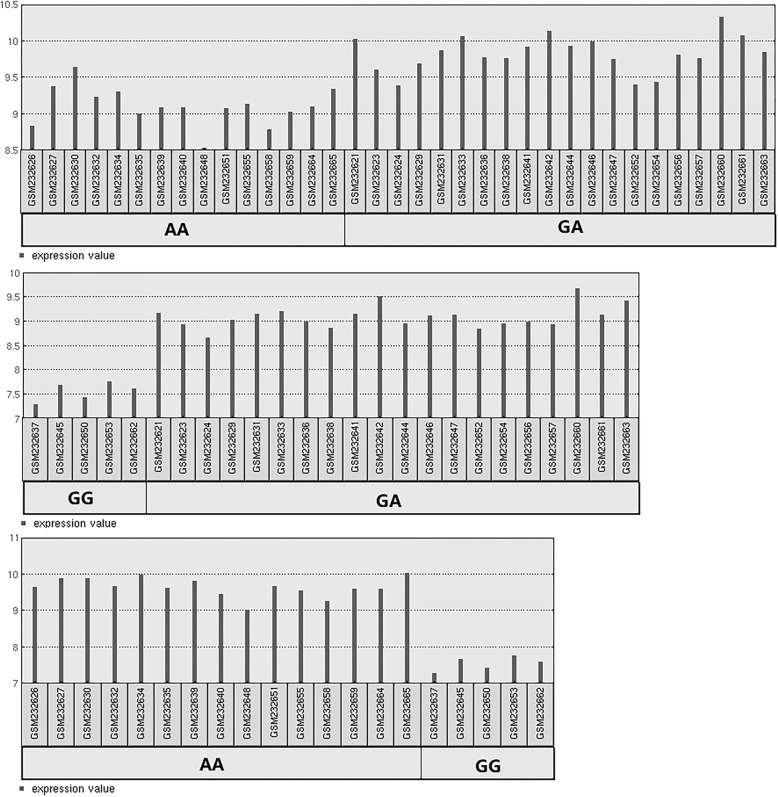
OAS1 mRNA expression levels among the different genotypes of rs10774671. The black bars represent fold change. The black bars below show all sample identifiers annotated by GSM numbers

eQTL analysis using the GTEx Portal database showed significant eQTL effects of rs10774671 (*P* < 0.001) on *OAS1* expression in whole blood from 369 individuals. The 40 CHB were divided into three groups according to rs10774671genotypic data. In brief, 15 CHB had the AA genotype, 20 had the GA genotype and 5 had the GG genotype. As shown in Fig. 2, GEO2R analysis revealed that the rs10774671 polymorphism was associated with *OAS1* expression levels after adjustment for multiple comparisons (*P* < 0.001).

## Discussion

In China, the ethnic Tibetan population is more likely to be infected with TB than the Han population [32, 33]. It is possible that host genetic difference is one of the bases of TB susceptibility*.* In this study, four SNPs in *OAS1* (rs2240190, rs1131454, 10,774,671 and 11,066,453) were investigated in Chinese Han and Chinese Tibetan patients with TB. Our data showed, for the first time, that the G allele and the GG genotype of rs10774671 in *OAS1* were associated with TB in the two populations.

OAS1 is an enzyme that plays an important role in innate antiviral defence [34]. This enzyme is activated by the presence of double-stranded or single-stranded RNA with a secondary structure and catalyses the oligomerisation of ATP into 2-5A [35]. These processes activate latent RNase L, which degrades viral and cellular RNA and blocks protein synthesis. In accordance with this function, human OAS1 mainly affects susceptibility to viral infections [35, 36]. Given the importance of the OAS1 protein, SNPs in the *OAS1* gene may affect susceptibility to infectious diseases. In the present study, we demonstrated that polymorphisms in *OAS1* were associated with TB. It is unclear why the heightened antiviral enzyme activity in individuals was associated with TB, but this association may be explained as follows. One explanation is that the major role of the OAS proteins is as immune regulators in innate immunity, although previous studies indicated that they also play important roles in other cellular functions. The role of OAS1 in immune-related diseases has been shown by a number of association studies [25, 26]. It is well known that the immune response against TB plays a critical role in the progression of *MTB* infection. Another explanation is that the *OAS1* gene affects susceptibility to TB through the type II IFN pathway [14, 15]. Type II IFN (IFN-γ) is critical for host defence against certain bacterial and parasitic pathogens [37]. IFN-γ can activate infected host macrophages to inhibit the replication of *MTB* directly [38]. In addition, OAS gene expression was regulated by both type I and type II IFNs [39]. A previous study suggested that IFN-γ could increase the levels of OAS mRNA [40]. Furthermore, *OAS1* gene up-regulation was observed in several gene expression signatures that differentiated active TB from latent TB infection [41, 42]. It was also established that there was a prominent correlation between OAS expression and TB [14]. Moreover, data from Noguchi et al. revealed the potential role of *OAS1* polymorphisms in respiratory infection [27]. Therefore, we assume that the polymorphism in the *OAS* gene may be associated with the risk of TB infection through the type II IFN pathway.

As a functional *OAS1* polymorphic marker, rs10774671 was reported to be associated with disease/viral infection [36, 43]. rs10774671 is located at the last nucleotide of intron 6 in *OAS1*, which acts as a splice-acceptor site for exon 7. The G allele was demonstrated to allow splicing to occur leading to the production of a p46 form with high enzymatic OAS activity [20]. The rs10774671 A allele usually prevents splicing at this site and thus splicing happens further downstream, resulting in the p48 and p52 forms associated with low OAS enzymatic activity. rs10774671 could also control other splice variants of OAS including p42 and p44 [20]. In addition, the HapMap genotypic data (Fig. 2) of rs10774671 suggested that individuals with GG genotype had lower expression levels than those with AA and GA genotypes. However, rs10774671 GG genotype was reported to be associated with the highest enzyme activity of OAS1 in unstimulated lymphocytes [44]. The inconsistent results between mRNA expression and enzyme activity may attribute to the reason that gene expressions at the transcriptional and translational levels might be different. Our data were partly consistent with these observations, in that the G allele and GG genotype had significant protective effects against TB, whereas the AG genotype was not associated with TB. Furthermore, this result was validated in the Chinese Han population. Considering the aforementioned functional effects of rs10774671 on phenotypes, we speculate that rs10774671 could alter *OAS1* expression levels precluding the type I IFN pathway resulting in a protective response.

In order to detect whether two or more SNPs interact either directly or indirectly to change TB risk separate from their independent effects, we conducted a gene-gene interaction analysis. We did not find any significant differences in single SNP analysis between cases and controls groups for rs2240190, rs1131454 and 11,066,453. However, rs2240190, rs1131454 and rs10774671 formed the best gene-gene interaction model. This result was consistent with the phenomenon that most diseases including TB are caused by multiple factors and gene-gene or gene-environment interactions are characteristic of the genetic factors. In addition, we also conducted a haplotype analysis which was based on the association between a polymorphism and the ancestral haplotype [45]. However, inconsistent with the gene-gene interaction result, there was no significant association between haplotypes and TB. Since the haplotype analysis approach was used for analyzing haplotype effects at numerous closely linked loci, and the gene-gene interaction analysis could be used for analyzing the unlinked loci [46, 47]. We speculate the discrepant results are likely attributable to different methods.

rs2240190 is located in intron 1 and it may have an alternative splicing regulatory effect, based on the Functional Single Nucleotide Polymorphism database [48]. To the best of our knowledge, no previous studies investigated the association between rs2240190 and disease. Only one study suggested that this genetic polymorphism was not in LD with rs10774671 [27]. rs1131454 (formerly rs3741981), in the evolutionarily conserved exon 3, is nonsynonymous (the G → A transition results in a G → S amino acid change). This polymorphic locus is close to the dsRNA binding domain of all OAS1 isoforms. In general, the AA, GA and GG genotypes of rs1131454 lead to low, intermediate and high OAS1 enzyme activity, respectively [44]. This polymorphic locus was demonstrated to be functional in the progression of the severe acute respiratory syndrome [49]. In our study, we demonstrated that the G allele and GG genotype were associated with TB in the Han population.

Genome-wide association studies with longitudinal data found that rs11066453 was a significant disease susceptibility locus, as confirmed by the Health Examination cohort [50]. Furthermore, the rs11066453 polymorphism was strongly related to *OAS1* mRNA expression levels in Epstein–Barr virus infection [51], and the monoallelically expressed *OAS1* gene was also significantly associated with the gamma-glutamyltransferase level [28]. This strong association of the *OAS1* gene that contains the rs11066453 polymorphism in the development and progression of human diseases may be due to it affecting *OAS1* expression. However, inconsistent with these findings, there was no significant association with TB susceptibility detected in the present study.

There are several limitations in our study. First, lack of data regarding the association between other genes in the OAS cluster and TB limited our understanding of genetic mechanisms regulating the pathogenesis of TB. Second, our study lacked clinical information for the Tibetans, which limited the analysis of the association with clinical characteristics. Finally, functional validation of the included SNPs was not carried out. As a result, the true causal allele underlying the genetic association result is still unknown.

In conclusion, we were the first to investigate the association between *OAS1* polymorphisms and TB in the Chinese Tibetan population and validated the results in an independent cohort. We found that rs10774671, located in the *OAS1* gene region is associated with susceptibility to TB in Tibetans and may act as a protective factor against TB, which was validated in the Chinese Han population. In addition, we also found that rs1131454 was a protective factor against TB in the Chinese Han population. rs2240190, rs1131454 and rs10774671 formed the best gene-gene interaction model in MDR analysis and may be considered as one of the multiple contributors to the progress of this complicated disease. However, in order to get more detailed mechanistic insight into the pathway of how OAS1 may act on TB, there is a need for further research in this field. Similar studies in different populations, gene-environment interactions analysis and functional studies are warranted to confirm and reinforce our results.

## Abbreviations

CI: Confidence interval
HIV: Human immunodeficiency virus
HWE: Hardy-Weinberg equilibrium
IFN: Interferon
iMLDR: Improved multiple ligase detection reaction
LD: Pairwise linkage disequilibrium
MAF: Minor allele frequency
MDR: Multifactor dimensionality reduction
MTB: *Mycobacterium tuberculosis*
OAS1: 2′-5′-oligoadenylate synthetase 1
OR: Odds ratio
RNase L: Ribonuclease L
RR: Relative risks
SNPs: Single-nucleotide polymorphisms
TB: Tuberculosis
